# Aerobic Exercise Training Prevents Heart Failure-Induced Skeletal Muscle Atrophy by Anti-Catabolic, but Not Anabolic Actions

**DOI:** 10.1371/journal.pone.0110020

**Published:** 2014-10-17

**Authors:** Rodrigo W. A. Souza, Warlen P. Piedade, Luana C. Soares, Paula A. T. Souza, Andreo F. Aguiar, Ivan J. Vechetti-Júnior, Dijon H. S. Campos, Ana A. H. Fernandes, Katashi Okoshi, Robson F. Carvalho, Antonio C. Cicogna, Maeli Dal-Pai-Silva

**Affiliations:** 1 Department of Morphology, São Paulo State University, Botucatu, São Paulo, Brazil; 2 Department of Internal Medicine, São Paulo State University, Botucatu, São Paulo, Brazil; 3 Department of Biochemistry, São Paulo State University, Botucatu, São Paulo, Brazil; 4 Centre of Biological and Health Sciences, North University of Paraná, Londrina, Paraná, Brazil; Rutgers New Jersey Medical School, United States of America

## Abstract

**Background:**

Heart failure (HF) is associated with cachexia and consequent exercise intolerance. Given the beneficial effects of aerobic exercise training (ET) in HF, the aim of this study was to determine if the ET performed during the transition from cardiac dysfunction to HF would alter the expression of anabolic and catabolic factors, thus preventing skeletal muscle wasting.

**Methods and Results:**

We employed ascending aortic stenosis (AS) inducing HF in Wistar male rats. Controls were sham-operated animals. At 18 weeks after surgery, rats with cardiac dysfunction were randomized to 10 weeks of aerobic ET (AS-ET) or to an untrained group (AS-UN). At 28 weeks, the AS-UN group presented HF signs in conjunction with high TNF-α serum levels; soleus and plantaris muscle atrophy; and an increase in the expression of TNF-α, NFκB (p65), MAFbx, MuRF1, FoxO1, and myostatin catabolic factors. However, in the AS-ET group, the deterioration of cardiac function was prevented, as well as muscle wasting, and the atrophy promoters were decreased. Interestingly, changes in anabolic factor expression (IGF-I, AKT, and mTOR) were not observed. Nevertheless, in the plantaris muscle, ET maintained high PGC1α levels.

**Conclusions:**

Thus, the ET capability to attenuate cardiac function during the transition from cardiac dysfunction to HF was accompanied by a prevention of skeletal muscle atrophy that did not occur via an increase in anabolic factors, but through anti-catabolic activity, presumably caused by PGC1α action. These findings indicate the therapeutic potential of aerobic ET to block HF-induced muscle atrophy by counteracting the increased catabolic state.

## Introduction

Cardiac dysfunction precedes heart failure (HF) and is typically characterized by enlargement of the heart and increased myocyte cell volume [1]. Pathological hypertrophy of the myocardium temporarily preserves its pump function, although prolongation of this state is a leading predictor of HF [1]. HF is the main cause of hospitalization and death worldwide. In the terminal phase of HF, a complex metabolic syndrome called cardiac cachexia, which is characterized by a loss of muscle and fat mass that is unresponsive to nutritional supplementation alone and which has been identified as a strong independent risk factor for mortality, is often observed [2]. However, the process leading to the progressive muscle atrophy that ultimately results in cardiac cachexia is still not completely understood.

Skeletal muscle atrophy is related to endocrine disorders and inflammatory conditions that can induce hypertrophy and atrophy-related gene/protein expression. Considerable evidence suggests that neurohormonal and immune mechanisms may play a central role in the pathogenesis of HF by controlling the balance between pro- and anti-growth signals in the skeletal muscle. Several factors have been demonstrated to be responsible for muscle wasting during HF, including reduced local insulin-like growth factor I (IGF-I) expression [3], increased muscular tumor necrosis factor alpha (TNF-α) [4], increased myostatin expression [5], and overexpression of the E3-ligases Muscle RING Finger 1 (MuRF-1) and atrogin-1/Muscle Atrophy F-Box (MAFbx), which activate the ubiquitin–proteasome system [6]. However, a direct effect of humoral factors on muscle-mass regulators during HF has not been well established.

In HF, alterations in multiple anabolic and catabolic systems result in progressive catabolism, which leads to skeletal muscle atrophy in advanced stages of the disease [7], [8]. IGF-I/AKT/mTOR is an essential regulator of skeletal muscle capacity for protein synthesis and degradation [9]. In the canonical IGF-I/AKT/mTOR pathway, AKT activation of mTOR and the subsequent phosphorylation of p70S6K and 4E-BP1 induce protein synthesis. In the early stages, chronic HF-related muscle atrophy is associated with reduced expression of IGF-I [10]. In addition to protein synthesis, the IGF-I pathway inhibits protein degradation through the sequestration of the forkhead box O (FoxO) family of transcription factors in the cytoplasm, where they are transcriptionally inactive [11]. In skeletal muscle, FoxO1 and 3 activation have been observed under conditions of muscle atrophy, such as cachexia [12] and HF [13], thereby promoting the transcription of atrophy-related genes. However, FoxO-induced muscle atrophy activity is also modulated by other intracellular signaling molecules, such as peroxisome proliferator-activated receptor-γ coactivator-1α (PGC1α), a regulator of skeletal muscle mass, particularly in conditions of muscle atrophy [14]. Significant evidence has demonstrated that increased PGC1α expression is sufficient to inhibit *in vivo* FoxO3-induced muscle fiber atrophy and that transgenic muscle-specific PGC1α overexpression protects against denervation- and fasting-induced atrophy, an effect associated with a reduction of the expression of MAFbx, MuRF1, and cathepsin L [14].

Exercise training (ET) is a widely accepted and widely proposed nonpharmacological intervention to minimize the symptoms of HF [15]. In humans, ET has been shown to directly improve metabolic and functional abnormalities of the peripheral muscles without changing cardiac performance [16]. In addition, ET has anti-inflammatory effects, with the potential to reduce local cytokine expression (e.g., TNF-α, IL-1β, and IL-6) [4] and increase the expression of anti-apoptotic factors (e.g., myostatin) [17], indicating that this type of intervention could partially reverse the catabolic state in skeletal muscle.

While the potential beneficial effects of ET on the loss of muscle mass in HF are clear, the exact molecular mechanisms by which ET might delay the onset of cardiac cachexia in the HF remain unclear. Therefore, the aim of the present study was to examine the effects of ET during the transition from cardiac dysfunction to HF on the expression of muscle mass-related anabolic and catabolic factors. We hypothesized that ET would increase anabolic factor expression and decrease catabolic factor expression, thus preventing muscle wasting during the transition from cardiac dysfunction to HF.

## Materials and Methods

### Animal Care and Experimental Design

All experiments and procedures were performed in accordance with the Guide for the Care and Use of Laboratory Animals published by the U.S. National Institute of Health and were approved by the Animal Ethics Committee, São Paulo State University (#198). Three- to four-week-old male Wistar rats weighing 90–100 g were anesthetized with a mixture of ketamine (50 mg/kg, i.p.) and xylazine (10 mg/kg, i.p.). A total of 16 rats was submitted to aortic stenosis (AS) surgery, which involved placing a 0.6-mm-i.d. stainless steel clip on the ascending aorta via a thoracic incision [1]. A total of 16 age-matched control animals underwent left thoracotomy without clip placement (Sham group). The rats were housed in collective polypropylene cages (2 animals per cage) covered with metallic grids in a temperature-controlled room (22–24°C) under a reverse light cycle (the dark cycle began at 7∶00 a.m., and the light cycle began at 7∶00 p.m.). The animals were given standard rat chow (Labina; Purina, SP, Brazil) and water *ad libitum*.

**Figure 1:**
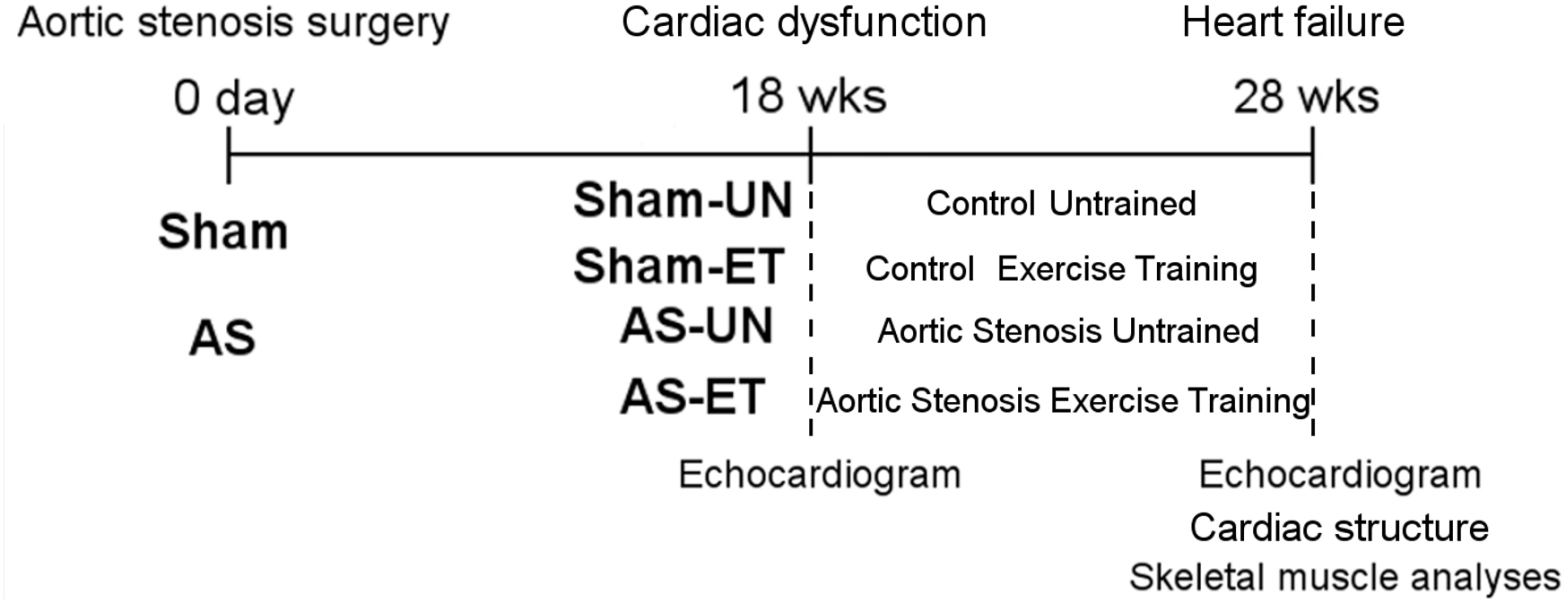
Experimental design. Sham, control group; AS, animals submitted to aortic stenosis surgery; Sham-UN, control untrained group; Sham-ET, control exercise training group; AS-UN, aortic stenosis untrained group; AS-ET, aortic stenosis exercise training group.

At 18 weeks after surgery, when the AS animals presented a cardiac dysfunction as measured by echocardiography, both groups (Sham and AS) were randomly redistributed in either sedentary untrained groups (Sham-UN, n = 8, and AS-UN, n = 8) and groups that underwent to 10 weeks of aerobic ET protocol (Sham-ET, n = 8, and AS-ET, n = 8). The ET protocol is described in detail below. Our laboratory has previously demonstrated that rats with AS start to exhibit evidence of HF approximately 21–25 weeks after surgery [18], [19]. Thus, after 28 weeks, all 4 groups underwent another echocardiogram and were then euthanized. The experimental design is shown in Figure 1. At the time of euthanasia, the rats were weighed and anesthetized with intraperitoneal pentobarbital sodium (50 mg/kg). The heart was removed via thoracotomy, and the atria and ventricles were separated and weighed. The soleus and plantaris muscles, which were chosen because of their contrasting structural and metabolic characteristics (i.e., fiber type distribution and prevalent metabolism), were harvested, rapidly frozen in isopentane cooled by liquid nitrogen, and subsequently stored at −80°C. For the HF examination, 2 observers determined the presence or absence of clinical and pathological HF features at the time of euthanasia. The clinical finding suggestive of HF was tachypnea and labored respiration. The pathological assessment of cardiac decompensation included left atrial thrombi, pulmonary congestion (lung weight/body weight ratio>2 SD above the mean for the Sham-UN group), right ventricular hypertrophy, ascites, and hepatic congestion [20]. In this study, rats with atrial thrombi, pulmonary congestion, and/or right ventricular hypertrophy were considered to have HF [21].

### Lactate Threshold Determination

To determine their lactate threshold and the velocity at which the lactate threshold occurred, the animals allocated into the exercise training study were subjected to incremental exercise testing on a motor treadmill adapted to experimental models [22]. The lactate threshold was defined as the running velocity that could be maintained without a lactate increase of 1.0 mmol/L above the blood lactate concentration obtained at the previous speed [23]. The protocol used for the incremental load test was adapted from that previously described by Carvalho et al. [24] and was performed at the end of Week 1 (week of adaptation), Week 4, and Week 7.

After a 1-week (10 min/day) adaption to the treadmill exercises, the rats were submitted to exercise testing. The treadmill speed started at 6 m/min and was progressively increased by 3 m/min every 3 min at 0% grade until exhaustion, which was defined as the point at which the rats could no longer maintain a running speed over 3 min. After each load increase, the animal was manually removed from the treadmill for 1 min for blood collection. Blood samples were taken from the tail vein and was pipetted evenly onto a test strip, which was inserted into a portable lactate analyzer (Accutrend Lactate, Boehringer-Mannheim) [25], [26]. The equipment was calibrated with a code strip specific to each lactate determination package.

### Aerobic Exercise Training

Rats in the training groups, Sham-ET and AS-ET, performed an aerobic ET program for 10 weeks (5 days/week). The program was modified from those previously described by De Souza et al. [27] and Siu et al. [28]. The duration and intensity of the training sessions progressively increased from 5 min, 5 m/min (Sham-ET and AS-ET, 1^st^ week) to 10, 12, and 14 min, 9 m/min (Sham-ET) and 6 m/min (AS-ET, 2^nd^ to 4^th^ week); 16, 18 and 20 min, 18 m/min (Sham-ET) and 12 m/min (AS-ET, 5^th^ to 7^th^ week); 20, 22 and 22 min, 21 m/min (Sham-ET) and 15 m/min (AS-ET, 8^th^ to 10^th^ week). The running speed corresponded to the lactate threshold, which was determined by the incremental exercise test (described above) performed at the end of Weeks 1, 4, and 7 to adjust the training velocities. The training sessions were performed at the same time of day, between 2 and 4 p.m.

### Echocardiographic Study

Echocardiographic measurements were performed before and after aerobic ET using a commercially available echocardiograph (General Electric Medical Systems, Vivid S6, Tirat Carmel, Israel) equipped with a 5–11.5 MHz multifrequency transducer. Rats were anesthetized by intramuscular injection of a mixture of ketamine (50 mg/kg) and xylazine (0.5 mg/kg) and positioned in supine position and ultrasound transmission gel was applied to the precordium. A two-dimensional parasternal short-axis view of the left ventricle (LV) was obtained at the level of the papillary muscles. M-mode tracings were obtained from short-axis views of the LV at or just below the tip of the mitral-valve leaflets, and at the level of the aortic valve and left atrium. All LV structures were manually measured by the same observer according to the leading-edge method of the American College of Echocardiography [29]. The measurements obtained were the mean of at least five cardiac cycles on the M-mode tracings. The following structural variables were measured: left atrium (LA) diameter, LV diastolic and systolic dimensions (LVDD and LVSD, respectively), LV diastolic posterior wall thickness (PWT), LV relative thickness in diastole (RWT; calculated as 2*PWT/LVDD), and aortic diameter (AO). Left ventricular function was assessed by the following parameters: heart rate (HR), endocardial fractional shortening (FS), LV ejection fraction (EF), posterior wall shortening velocity (PWSV), and early-to-late diastolic mitral inflow ratio (E/A ratio).

### Muscles and Histochemical Procedures

**Figure 2:**
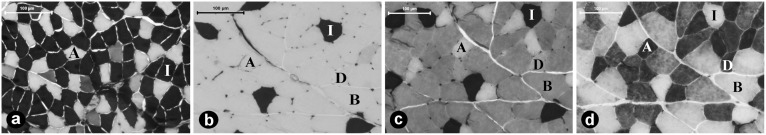
Serial cross sections of the rat hindlimb muscles. Soleus (a) and plantaris (b, c and d) muscles taken from a Sham-UN group rat demonstrating fiber-type delineation as determined by the myofibrillar adenosine triphosphatase (mATPase) reaction after preincubation at pH 4.2 (b), 4.5 (a, c), and 10.6 (d). (I, type I; A, type IIA; D, type IID; and B, type IIB).

Serial histological sections (12 µm thick) from soleus and plantaris muscles were obtained in a cryostat (JUNG CM1800, Leica Germany) at −24°C to histochemical analysis. To determine muscle fiber-type frequency and cross sectional area (CSA) myofibrillar adenosine triphosphatase (mATPase) histochemistry was performed after preincubation at pH 4.2, 4.5 and 10.6 [30]. Muscle fibers (Type I, IIA, IID, and IIB) were identified based on their staining intensities [31] (Figure 2). The stained sections were used for photographic documentation of 4 histological fields (400 random fibers; 20×lens) from soleus and plantaris muscles of each animal. Fiber type frequency and CSA (used as an index of type-specific fiber atrophy) were analyzed using an Image Analysis System Software (Leica QWin Plus, Germany).

### Serum TNF-α and IGF-I Levels

TNF-α and IGF-I levels were analyzed by enzyme-immunometric using a commercially available kits (Quantikine Rat TNF-α Immunoassay, and Quantikine Mouse/Rat IGF-I Immunoassay, R & D Systems, Minneapolis, MN) and the procedures were performed according to manufacturer’s instructions. All the samples were run in duplicate and the average values are reported.

### Biochemical Assay Procedures

Soleus and plantaris muscle samples (200 mg) were homogenized in 5 ml cold phosphate buffer (0.1 mmol/L, pH 7.4) using a motor-driven Teflon glass Potter-Elvehjem (1 min, 100×g). The whole homogenate was centrifuged at 10,000×g for 15 min, and the supernatant was used for determination of lactate dehydrogenase (LDH; E.C.1.1.1.27) and citrate synthase (CS; E.C.4.1.3.7). CS was measured in a medium containing 50 mmol/L Tris-HCl pH 8.1, 0.3 mmol/L acetyl-CoA, 0.1 mmol/L DTNB (5,5′ dithiobia-2-nitrobenzoic acid), and 0.5 mmol/L oxaloacetate, according to Bass and colleagues [32]. The assay medium for LDH contained 50.0 mmol/L Tris-HCl buffer pH 7.5, 0.15 mmol/L nicotinamide adenine dinucleotide reduced form (NADH), and 1 mmol/L pyruvate, as previously described [33]. Enzyme activities were performed at 25°C using a microplate reader (µQuant-MQX 200 with Kcjunior software to computer system control, Bio-Tec Instruments, Winooski, Vermont, USA). All chemicals and solvents were from Sigma (St. Louis, Missouri, USA).

### Quantitative Real-time PCR

Total RNA was extracted from soleus and plantaris muscles using Trizol Reagent (Life Technologies, CA, USA), solubilized in nuclease-free water and quantified (NanoVue Plus; GE HealthCare, Little Chalfont, UK). After assessment of RNA concentration (ng/µl), purity (ensured by 260/280 nm ratio of ∼2.0) and integrity (RNA integrity number >8; RNA 6000 Nano assay; Agilent, Waldbronn, Germany), the RNA was treated with Amplification Grade Deoxyribonuclease I (Life Technologies, Carlsbad, CA, USA) to remove any genomic DNA contamination. cDNA was synthesized using High Capacity cDNA Reverse Transcription Kit (Life Technologies, Carlsbad, CA, USA) according to the manufacturer’s protocols, and the genes involved in atrophic system, as TNF-α (Rn00562055_m1), NFκB (Rn01502266_m1), MAFbx (Rn00591730_m1), MuRF1 (Rn00590197_m1), FoxO1 (Rn01494868_m1) and myostatin (Rn00569683_m1); hypertrophic system IGF-I (Rn00710306_m1), AKT1 (Rn00583646_m1) and mTOR (Rn00571541_m1) and anti-proteolytic PGC1α (Rn00580241_m1) were analyzed. All genes were measured by real-time quantitative PCR (RT-qPCR) using microfluidic TaqMan Low Density Array (TLDA; Life Technologies, Carlsbad, CA, USA) cards. Five control genes were tested 18S rRNA (Hs99999901_s1), Actb (Rn00667869_m1), Gapdh (Rn01775763_g1), Hprt1 (Rn01527840_m1) and Tbp (Rn01455648_m1) and Hprt1, Actb and Tbp were used as reference genes to normalize the data as they recorded the highest stability. A total of 100 µl reaction mixture with 50 µl of cDNA template (200 ng mRNA) was added to 50 µl of TaqMan Universal PCR Master Mix (Applied Biosystems, Foster City, CA, USA) and was dispensed into loading wells on the TLDA card. The cards were centrifuged twice at 1,200 rpm for 1 min each time, sealed, and placed in the thermal cycler. The following cycling conditions were used for all TLDA applications: 50°C for 2 min, 95°C for 10 min, and 40 cycles of 95°C for 15 s followed by 60°C for 1 min and thermal cycling and fluorescence detection was performed on Applied Biosystems ABI Prism ViiA7 Sequence Detection System with ABI Prism ViiA7 SDS Software 2.4. TLDAs data were analyzed using the DataAssist software 2.0 (Life Technologies).

### Western blot

Protein levels of TNF-α, NFκB (p65), MAFbx, MuRF1, FoxO1, Phospho-FoxO1 (Ser^256^), myostatin, IGF-I, AKT, Phospho-AKT (Ser^473^), mTOR, Phospho-mTOR (Ser^2448^), PGC1α and Gapdh were quantified by Western blot assays in soleus and plantaris muscle extracts. Muscle samples were homogenised in lysis buffer (1% Triton X-100, 10 mM sodium pyrophosphate, 100 mM NaF, 10 µg/mL aprotinin, 1 mM phenylmethylsulfonylfluoride (PMSF), 0.25 mM Na_3_VO_4_, 150 mM NaCl and 50 mM Tris-HCl pH 7.5). The samples were centrifuged and 50 µL of the homogenate fraction were resuspended in 25 µL of Laemmli loading buffer (2% SDS, 20% glycerol, 0.04 mg/mL bromophenol blue, 0.12 M Tris-HCl pH 6.8, and 0.28 M b-mercaptoethanol). Fifty micrograms of total protein were separated by one-dimensional SDS-PAGE, stained with Ponceau S red (Sigma Chemical) to confirm equal loading of each sample. As a second approach to verify similar loading between the lanes, gels were loaded in duplicate, and one gel was stained with Coomassie blue. Proteins were transferred from a gel to a nitrocellulose membrane (Bio-Rad Laboratories, Hercules, CA, USA). Using a vacuum-enhanced detection system (SNAP i.d., Millipore, Billerica, MA, USA) nonspecific binding sites were blocked with a 3% bovine serum albumin (BSA) solution in phosphate-saline buffer (PBS-T: 0.1 M NaH_2_PO_4_·H_2_O, 0.1 M Na_2_HPO_4_·7H_2_O, 0.15 M NaCl, 0.1% Tween-20, pH 7.4) for 10 minutes. Following blocking, the membranes were incubated with specific primary antibodies against TNF-α (Santa Cruz, California, USA; #sc-1349), NFκB - p65 (Santa Cruz, California, USA; #sc-8008), MAFbx (Santa Cruz, California, USA; #sc-27644), MuRF1 (Santa Cruz, California, USA; #sc-27642), FoxO1 (Cell Signaling, Beverly, USA; #9454), myostatin (Santa Cruz, California, USA; #sc-6884), Phospho-FoxO1 (Cell Signaling, Beverly, USA; #9461), IGF-I (Santa Cruz, California, USA; #sc-7144), AKT (Cell Signaling, Beverly, USA; #9272), Phospho-AKT (Cell Signaling, Beverly, USA; #4060), mTOR (Cell Signaling, Beverly, USA; #2972), Phospho-mTOR (Cell Signaling, Beverly, USA; #5536), PGC1α (Abcam, Cambridge, UK; ab-106814) and Gapdh (Cell Signaling, Beverly, USA; #2118) in a 1% BSA solution for 10 min. After four wash steps with PBS-T, membranes were incubated in a 1∶2,000 dilution of specific secondary antibodies (Santa Cruz Biotechnology, Santa Cruz, CA, USA) conjugated with horseradish peroxidase for 10 min. Finally, immunoreactive protein signals were detected using SuperSignal West Pico Chemiluminescent Substrate Kit (Thermo Fisher Scientific, Rockford, IL, USA), according to the manufacturer’s instructions. The chemiluminescent signal was visualized and quantified by densitometry using the image analyzer ImageQuant 350 (GE Healthcare, Little Chalfont, UK). The values were normalized by the values obtained for Gapdh protein.

### Statistical Analysis

Data are expressed as mean ± SD. Shapiro-Wilk normality test was used to verify data normal distribution. Differences in echocardiographic parameters before aerobic ET between Sham and AS animals were tested with unpaired Student’s t test. The muscle fiber type frequency data were analyzed using Kruskal-Wallis test. One-way analysis of variance (ANOVA) followed by *a posteriori* Tukey multiple comparison test was used to analyze circulating TNF-α, CSA, CS, LDH, mRNA and protein expression data after ET among the groups. To analyze the involvement of exercise on cardiac parameters between before and after aerobic ET, echocardiographic values were probed with two-way ANOVA for repeated measures with a Bonferroni’s *post test*. Linear regression was used to assess the relationship between PGC1α - FoxO1 mRNA and protein expression in the plantaris muscle. Statistical significance was considered achieved when the value of p was <0.05.

## Results

### Clinical and Anatomical Parameters

Relevant anatomical data are summarized in Table 1. At 18 weeks post-surgery and before the aerobic ET period, all the AS rats displayed deterioration of cardiac structure and function but did not present HF signs. In this study, an HF diagnosis was based on the presence of tachypnea and labored respiration and confirmed by pathological *post mortem* findings, such as right ventricular hypertrophy, pulmonary congestion, left atrial thrombus, and ascites. At 28 weeks after surgery, six of 8 AS-UN animals showed severe ascites, moderate tachypnea and atrial thrombi; however, the AS-ET animals presented a reduced intensity and frequency of these signs. Of the 8 AS-ET rats, three had mild ascites and tachypnea and only one presented atrial thrombi. Although the LV weight and LV/BW ratio were increased in both of the AS groups, the ET period attenuated the presence of cardiac thrombi and the increase in the atria, right ventricle, and lung weights in both absolute and BW-normalized values.

**Table 1:** Anatomical data after exercise training.

	Sham-UN (n = 8)	Sham-ET (n = 8)	AS-UN (n = 8)	AS-ET (n = 8)
BW (g)	489±29	475±48	464±36	440±45
LV (g)	0.77±0.07	0.65±0.07	1.15±0.15*	1.06±0.13#
LV/BW (mg/g)	1.57±0.18	1.37±0.20	2.53±0.23*	2.45±0.22#
RV (g)	0.25±0.05	0.24±0.03	0.46±0.10*	0.35±0.09
RV/BW (mg/g)	0.52±0.09	0.51±0.04	1.00±0.26*	0.86±0.25
Atria (g)	0.11±0.03	0.10±0.01	0.40±0.08*	0.28±0.09^#^ †
Atria/BW (mg/g)	0.23±0.06	0.20±0.02	0.87±0.17*	0.64±0.22^#^ †
Incidence of cardiac thrombi	0%	0%	56%	11%
Lung (g)	1.92±0.44	1.89±0.20	3.25±0.62*	2.52±0.59†
Lung/BW (mg/g)	3.92±0.93	4.00±0.54	7.16±1.53*	5.49±1.17†

Sham and aortic stenosis rats that remained untrained (UN) or were submitted to aerobic exercise training (ET) during the 10 weeks of training protocol; AS-ET, aortic stenosis rats that BW, body weight; LV, left ventricle; RV, right ventricle. Data are expressed as mean ± SD, except for the percentage of animals that displayed cardiac thrombi.

*p<0.05 vs. Sham-UN;

# p<0.05 vs. Sham-ET;

† p<0.05 vs. AS-UN.

### Cardiac Geometry, Function and Running Performance

To evaluate cardiac function, all the animals were submitted to an echocardiographic study before and after the aerobic ET protocol (Table 2). Before ET, AS groups showed pathological cardiac remodeling evidenced by increased PWT and RWT, while cardiac dysfunction was demonstrated by a decrease in PWSV and an elevation of the mitral E/A wave and LA/AO ratios. No change in heart rate (HR) under anesthesia was observed, indicating that the depth of anesthesia was similar among the groups during the echocardiographic examination. At the end of the experiment, the AS-UN group displayed a deterioration of cardiac dysfunction compared with the AS group at 18-weeks as shown by significant LV dilation at systole (LVSD) and diastole (LVDD) and accompanied by an increased PWT and E/A wave ratio. Furthermore, after 10 weeks of the training period, the left ventricular PWSV, endocardial FS and EF deteriorated even further in the AS-UN group compared with the preprotocol 18-week AS rats.

**Table 2:** Echocardiografic data before and after exercise training.

	Before exercise training (18 wks)				After exercise training (28 wks)			
	Sham-UN	Sham-ET	AS-UN	AS-ET	Sham-UN	Sham-ET	AS-UN	AS-ET
HR, bpm	290±29	294±30	272±26	296±40	301±26	296±31	282±49	282±55
LVDD, mm	8.13±0.42	8.05±0.51	8.31±0.57	8.15±0.87	8.40±0.66	8.42±0.38	9.14±0.32* ‡	8.19±0.77†
LVSD, mm	3.49±0.59	3.72±0.55	3.91±0.73	3.45±0.62	3.82±0.63	3.84±0.36	5.08±0.57* ‡	3.41±1.01†
PWT, mm	1.45±0.11	1.42±0.04	2.09±0.16*	2.02±0.24#	1.46±0.06	1.43±0.07	2.29±0.20* ‡	2.15±0.25#
RWT	0.36±0.04	0.36±0.03	0.48±0.05*	0.52±0.04#	0.35±0.03	0.34±0.02	0.51±0.05*	0.53±0.08#
LA/AO	1.43±0.16	1.50±0.17	1.93±0.36*	1.85±0.14#	1.44±0.10	1.56±0.18	2.21±0.26*	2.08±0.24#
FS, %	57.27±5.73	53.70±5.81	53.03±7.94	55.93±11.29	55.33±4.40	54.37±4.02	43.78±6.68* ‡	58.84±10.72†
PWSV,mm/s	40.40±2.62	38.61±5.56	25.93±2.89*	28.75±7.38#	37.72±4.81	37.17±3.51	23.30±3.91* ‡	29.18±5.17^#^ †
E/A	1.49±0.23	1.44±0.32	5.15±1.95*	4.97±1.36#	1.33±0.32	1.36±0.23	7.93±1.75* ‡	4.24±2.23^#^ †
EF, %	92±3	90±4	89±6	90±8	90±3	90±3	82±6* ‡	92±6†

HR, heart rate; LVDD and LVSD, LV diastolic and systolic diameters, respectively; PWT, LV posterior wall thickness; RWT, LV relative wall thickness in diastole; LA, left atrium diameter; AO, aortic diameter; FS, LV endocardial fractional shortening; PWSV, LV posterior wall shortening velocity; E/A, early-to-late diastolic mitral inflow ratio; EF, ejection fraction. n = 8 per group. Data are expressed as mean ± SD.

*p<0.05 vs. Sham-UN and,

# p<0.05 vs. Sham-ET at the same time (18 wk or 28 wk);

† p<0.05 vs. AS-UN;

‡ p<0.05 vs. AS; t test, one-way or 2-way ANOVA with Bonferroni *post hoc* test as appropriate.

**Figure 3:**
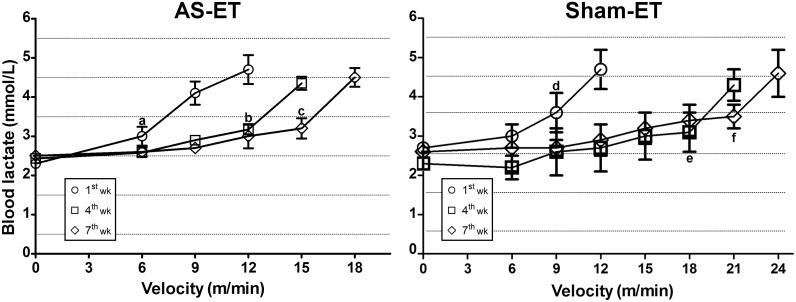
Blood lactate concentration in the AS-ET and Sham-ET groups assessed on the 1^st^, 4^th^ and 7^th^ weeks by incremental load test. Values are expressed as the mean ± SD. Significant differences (p<0.05) at the same time were in AS-ET group: a. vs. 9 m/min; b. vs. 15 m/min; and c. vs. 18 m/min. Sham-ET: d. vs. 12 m/min; e. vs. 21 m/min; and f. vs. 24 m/min.

The echocardiographic parameters remained unchanged in the AS-ET group after aerobic ET. Moreover, in the AS-ET the ET protocol prevented contractile dysfunction (decreased FS) and ejection fraction (EF) and avoided increases in the LV diameter at systole and diastole compared with the AS-UN group. In addition, we observed improved exercise tolerance and performance, as demonstrated by a significant increase in velocity at the lactate threshold in the Sham-ET and AS-ET groups between the Week 1 and Week 7 tests (Figure 3).

### Citrate Synthase and Lactate Dehydrogenase Activities

**Figure 4:**
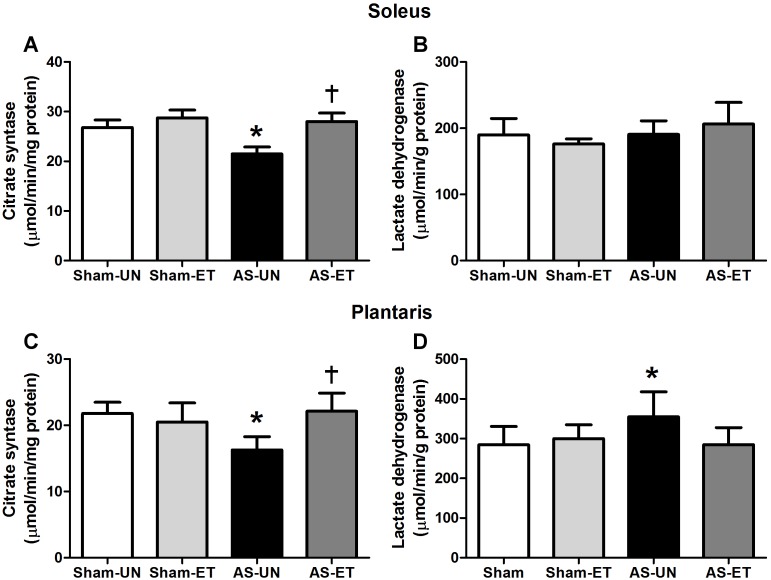
CS and LDH activities. Soleus (A and B) and plantaris (C and D) muscles of the Sham-UN, Sham-ET, AS-UN, and AS-ET groups. Data are presented as the mean ± SD. *p<0.05 vs. Sham-UN; †p<0.05 vs. AS-UN.

The activities of the enzymes citrate synthase (CS) (an indicator of cellular aerobic metabolism) and lactate dehydrogenase (LDH) (an indicator of anaerobic metabolism) were measured in soleus and plantaris muscle samples obtained from all groups after aerobic ET. HF induced a significant decrease (25%) in CS activity in both muscles in the AS-UN group compared with the Sham-UN group. CS maximal activity was similar within ET rats studied (Sham-ET and AS-ET), moreover, CS activity was preserved in AS-ET group when compared with AS-UN, suggesting that oxidative metabolism was improved, enhancing the muscle performance (Figure 4, A and C). LDH activity in the plantaris muscle was significantly increased in the AS-UN group compared with the Sham-UN group (Figure 4D), while the AS-ET group remained unchanged. In the soleus muscle, no significant difference was observed in LDH enzyme activity among the groups (Figure 4B).

### Muscle Fiber Type Frequency and Cross-Sectional Area

**Figure 5:**
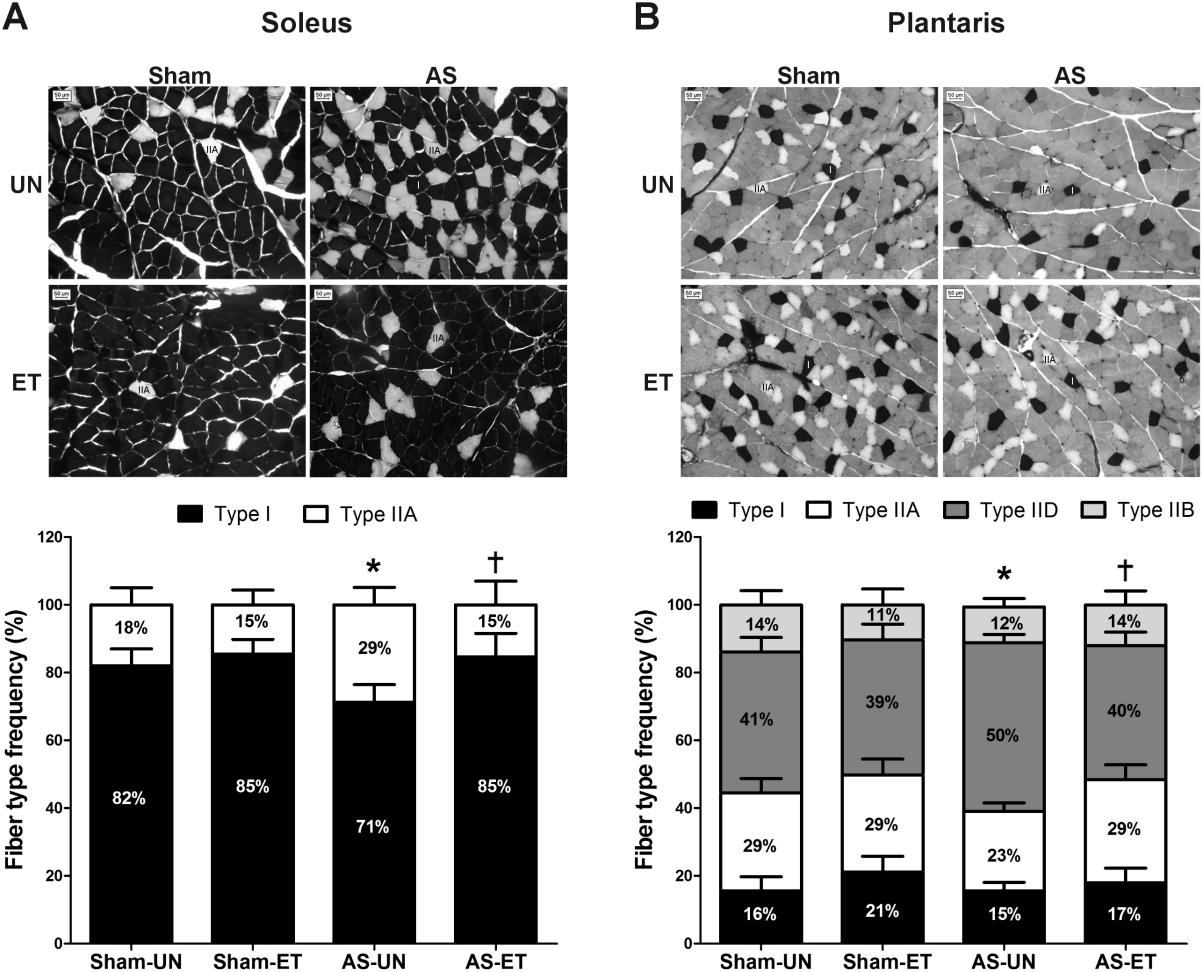
Soleus (A) and plantaris (B) muscle fiber-type frequencies. Representative photographs of mATPase reaction after preincubation at pH 4.5 and the data of the muscle fiber-type frequencies in Sham-UN, Sham-ET, AS-UN, and AS-ET groups. (Type I; type IIA; type IID; and type IIB). Soleus (A), type I and IIA *p<0.05 vs. Sham-UN; †p<0.05 vs. AS-UN. Plantaris (B), type IIA and IID, *p<0.05 vs. Sham-UN; †p<0.05 vs. AS-UN.

Using the histochemical reaction of myofibrillar ATPase (m-ATPase), we observed that aerobic ET performed between cardiac dysfunction and HF prevented in 16% the reduction in the percentage of slow-twitch fibers (i.e., Type I) and Type I-to-Type IIA fiber conversion in the soleus muscle of the AS-ET rats (Figure 5A). In the plantaris muscle, ET attenuated the decrease in Type IIA fibers to approximately 22%, and it decreased the Type IIA-to-Type IID fiber conversion in AS-ET compared to AS-UN group (Figure 5B). To determine if the aerobic ET subjected between cardiac dysfunction and HF was able to prevent atrophy of the soleus and plantaris muscles, we used the m-ATPase reaction to measure the skeletal muscle fiber cross-sectional area (CSA). At the end of the experiment, the AS-UN group presented a significant reduction in the CSA of Type I and IIA fibers in the soleus muscle (Table 3). Moreover, in this muscle, aerobic ET attenuated the reduction of fibers CSA in AS-ET animals. On the other hand, Type I and IIA fibers of plantaris muscle were not affected by AS and ET stimuli. However, a significant decrease in the CSA of plantaris Type IID and IIB fibers was observed in the AS-UN group, indicating that ET prevented the atrophy of plantaris Type IID and IIB fibers (Table 4).

**Table 3:** Cross sectional area (µm^2^) of the two major soleus muscle fiber types after ET.

	Sham-UN (n = 8)	Sham-ET (n = 8)	AS-UN (n = 8)	AS-ET (n = 8)
I	3564.09±307.87	3582.96±204.80	3056.92±281.86*	3565.18±376.28†
IIA	3485.77±232.03	3755.20±359.61	2950.67±223.70*	3976.28±408.96†

Data are expressed as mean ± SD.

*p<0.05 vs. Sham-UN;

† p<0.05 vs. AS-UN.

One-way ANOVA + Tukey test.

**Table 4:** Cross sectional area (µm^2^) of plantaris muscle fiber types after ET.

	Sham-UN (n = 8)	Sham-ET (n = 8)	AS-UN (n = 8)	AS-ET (n = 8)
I	2317.28±247.10	2223.10±231.19	2270.52±426.69	2158.09±378.18
IIA	2229.72±165.23	2222.16±186.57	2141.53±298.37	2246.69±282.28
IID	2703.84±272.88	2716.39±184.22	2307.12±276.74*	2679.91±303.61†
IIB	4159.27±397.15	4357.31±405.12	3508.91±228.85*	4273.55±490.45†

Data are expressed as mean ± SD.

*p<0.05 vs. Sham-UN;

† p<0.05 vs. AS-UN.

One-way ANOVA + Tukey test.

### Systemic Concentration of TNF-α and IGF-I

**Figure 6:**
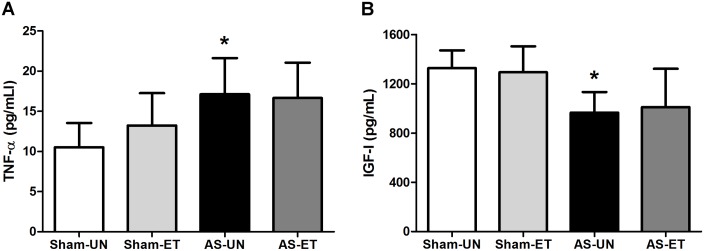
Serum concentration. Proinflammatory cytokine TNF-α (A) and growth factor IGF-I (B) in AS-UN, AS-ET, Sham-UN, and Sham-ET groups. Data are presented as the mean ± SD. *p<0.05 vs. Sham-UN.

The serum concentration of TNF-α showed a significant increase only between the non-trained HF animals (AS-UN group) and Sham-UN group (AS-UN: 18.04±3.96 pg/mL vs. Sham-UN: 12.07±1.67 pg/mL; p<0.05). However, AS-ET group still remained not significantly lower than of the AS-UN group (Figure 6A). On the other hand, the serum concentration of IGF-I was significantly lower in the non-trained rats (AS-UN group) as compared to Sham-UN (AS-UN: 918.7±123.2 pg/mL vs. Sham-UN: 1328.0±143.7 pg/mL, p<0.05); the trained rats (AS-ET) remained not significantly higher than the sedentary rats (Figure 6B).

### Skeletal Muscle Gene and Protein Expression

#### Proteolytic system

**Figure 7:**
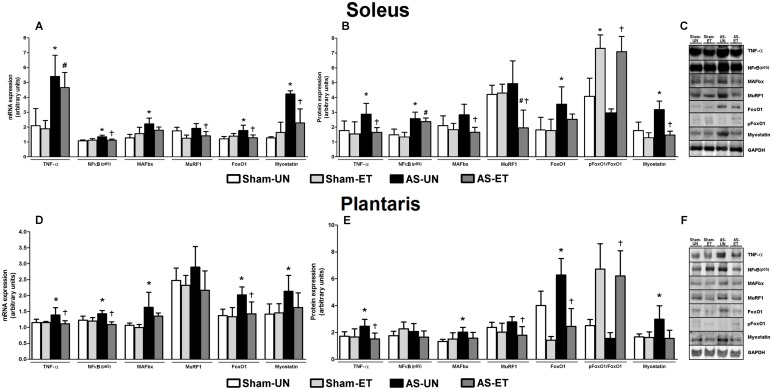
mRNA and protein levels of the skeletal muscle proteolytic system. A and D: mRNA levels in the soleus (A) and plantaris (D) muscles; protein content in the soleus (B) and plantaris (E) muscles; representative blots of soleus (C) and plantaris (F) muscles in Sham-UN, Sham-ET, AS-UN, and AS-ET groups. Data are expressed as the mean ± SD; *Significant difference from the Sham-UN group, p<0.05; #p<0.05 vs. Sham-ET; †p<0.05 vs. AS-UN group.

To investigate the effects of ET on changes in the skeletal muscle proteolytic system in HF animals, we measured the mRNA and protein levels of TNF-α, NFκB (p65), MAFbx, MuRF1, FoxO1, and myostatin in the soleus and plantaris muscles (Figure 7). Furthermore, the catabolic effect of HF on skeletal muscle was assessed by the phosphorylation status of FoxO1^Ser256^ in Sham-UN, Sham-ET, AS-UN and AS-ET rats. In the soleus muscle, there was a significant (p<0.05) increase in TNF-α, NFκB (p65), FoxO1, and myostatin gene (Figure 7A) and protein (Figure 7B and C) expression in the AS-UN group compared to the Sham-UN group (Figure 7B and C). Interestingly, this upregulation in catabolic factor expression was attenuated in the AS-ET group (Figure 7A and B) in conjunction with an increase in the phosphorylation levels of FoxO1^Ser256^. In addition, MAFbx mRNA was significantly increased, and the increases in MAFbx and MuRF1 protein content in the AS-UN group were significantly attenuated in the AS-ET group (Figure 7B and C). In the plantaris muscle (Figure 7D, E, and F), we observed a significant (p<0.05) increase in TNF-α and FoxO1 gene and protein expression in the AS-UN group and a decrease in the ratios of phosphorylated FoxO1 protein to total protein expression and a significant attenuation in the AS-ET group. Furthermore, a significant increase in myostatin gene and protein expression was observed in the AS-UN group, and attenuation of this increase at the protein level was observed in the AS-ET group. In addition, MAFbx gene and protein expression were increased in the AS-UN group compared with the Sham-UN group, but no significant effect of ET was observed in the AS-ET group. NFκB (p65) mRNA expression was increased in the AS-UN group and attenuated in the AS-ET group. Moreover, AS-UN group showed not significantly higher in MuRF1 gene and protein expression and attenuated protein levels in the AS-ET group.

#### Hypertrophy Pathway

**Figure 8:**
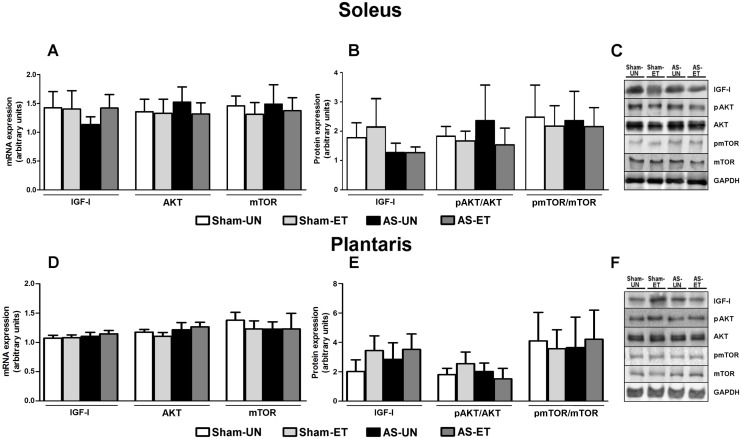
mRNA and protein levels of skeletal muscle hypertrophy components. A and D: mRNA levels in the soleus (A) and plantaris (D) muscles; protein content in the soleus (B) and plantaris (E) muscles; representative blots of soleus (C) and plantaris (F) muscles from Sham-UN, Sham-ET, AS-UN, and AS-ET groups. Data are expressed as the mean ± SD; *Significant difference from the Sham-UN group, p<0.05; #p<0.05 vs. Sham-ET; †p<0.05 from the AS-UN group.

To determine if ET stimulates the hypertrophic pathway and attenuates the atrophic effect observed in skeletal muscles in our HF model, we measured IGF-I expression and its downstream AKT and mTOR. Our results indicated that there were no differences (p>0.05) in IGF-I mRNA and protein levels in the soleus (Figure 8A and B) and plantaris (Figure 8D and E) muscles among the groups. Similarly, no changes in AKT and mTOR mRNA and the ratios of phosphorylated protein to total protein expression occurred among the groups in either muscle type (Figure 8).

#### Anti-Proteolytic Component

**Figure 9:**
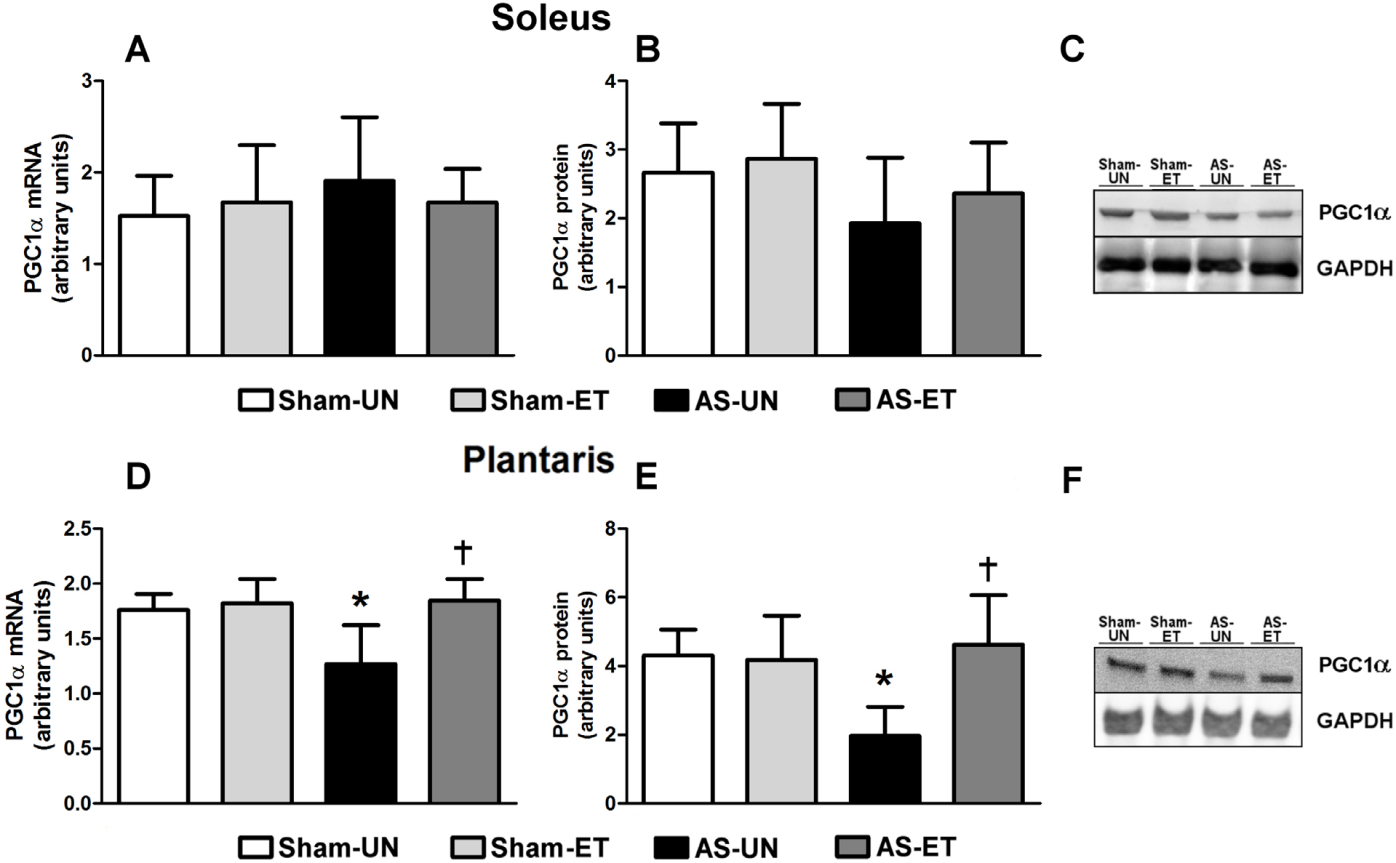
mRNA and protein levels of the skeletal muscle proteolysis inhibitor PGC1α. A and D: mRNA levels in the soleus (A) and plantaris (D) muscles; protein content in the soleus (B) and plantaris (E) muscles; representative blots of soleus (C) and plantaris (F) muscles in Sham-UN, Sham-ET, AS-UN, and AS-ET groups. Data are presented as the mean ± SD; *Significant difference from the Sham-UN group, p<0.05; †p<0.05 from the AS-UN group.

**Figure 10:**
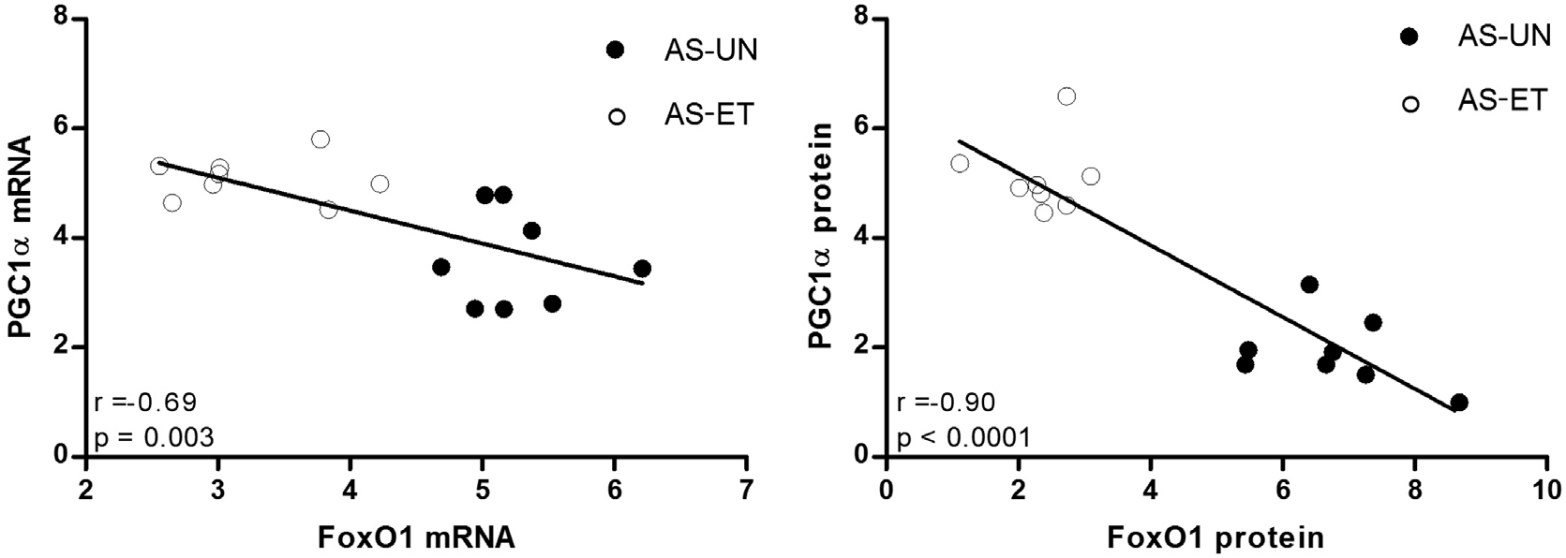
Relationship between PGC1α - FoxO1 mRNA and protein expression in the plantaris muscle. There were significant negative correlations between the PGC1α and FoxO mRNA/protein levels. For all correlations, individual data points from the AS-UN and AS-ET groups (n = 8 rats per group) were used.

To explore additional aspects of the prevention of skeletal muscle loss in HF via ET, PGC1α expression was investigated. As shown in Figure 9, although no statistically significant differences were observed in the soleus muscle, in the plantaris muscles, we observed a decrease in PGC1α mRNA levels in approximately 62% of the AS-UN animals (Figure 9D); furthermore, PGC1α protein levels were approximately 55% lower in the AS-UN animals compared with the other groups (Figure 9E and F). Interestingly, the AS-ET rats displayed significant attenuation of the reduction in PGC1α mRNA and protein levels, which remained at the same levels as in the Sham-ET group. In addition, linear regression analysis of the plantaris muscle results revealed a significant negative correlation between FoxO1 and PGC1α mRNA expression (Pearson’s *r* = −0.69, p = 0.003) and FoxO1 and PGC1α protein levels (Pearson’s *r* = −0.90, p<0.0001), based on an analysis of the individual data points for the AS-UN and AS-ET groups (Figure 10).

## Discussion

Metabolic abnormalities resulting in a progressive catabolic state have been observed in various chronic diseases [34], [35]. Since skeletal muscle dysfunction and loss of lean muscle mass have been described in HF [36], [37], a number of studies have focused on identifying therapies that could improve clinical outcomes. Studies of HF have reported that ET is an effective non-pharmacological therapy for improving exercise capacity and quality of life and confers survival benefits [38]. Moreover, recent reports have demonstrated that ET has beneficial effects against HF-induced skeletal myopathy [39], [40]. However, the molecular mechanisms by which ET attenuates or reverses skeletal muscle myopathy, which would become attractive targets for heart failure therapy, remain elusive.

In the present study, we used the ascending aortic stenosis (AS) model to promote a gradual development of left ventricular hypertrophy and posterior HF in rats [1]. In this model, animals develop cardiac remodeling that is associated, in the short term, with diastolic dysfunction and improved systolic function, followed by depressed systolic performance and HF. After 18 weeks of supra-aortic stenosis and before the training intervention, cardiac remodeling was noted on an echocardiographic assessment, although the animals did not show signs of HF. After 10 weeks of aerobic ET, the AS-ET animals presented an improved in structure and function in the systole and diastole compared with AS-UN. In addition, ET prevented the deterioration of cardiac structure and function in AS animals, when we compared the AS-ET group and the 18-week AS rats. Our data support previous findings that ET had a beneficial effect on the left ventricular function of individuals with HF [41], [42] and indicates that ET can be an important clinical intervention for improving cardiac structure and function during the transition from cardiac dysfunction to HF.

Animal [10] and human [39] studies have demonstrated skeletal muscle abnormalities in HF, including changes in morphology, function, and depressed skeletal muscle aerobic capacity. In our model of HF, we also observed changes in oxidative and glycolytic skeletal muscle metabolic enzymes. The reduction of CS enzyme activity in both the soleus and plantaris muscles in the AS-UN group, suggests a decrease in aerobic metabolism. After 10 weeks of aerobic ET, a significant attenuation in the decreasing CS activity in AS-ET rats was evident. Although, our aerobic ET protocol did not change this enzyme in the Sham-ET group, probably due to the low training intensity used, CS was clearly detected in the AS-ET animals. This prevention in the reduction of CS activity in the AS-ET group may participate in de novo ATP synthesis during ET in HF rats; therefore, the higher citrate synthase activity in AS-ET rats than in AS-UN rats suggests aerobic metabolism improvements. Furthermore, we observed an increase in LDH enzyme in the AS-UN group, indicating higher anaerobic metabolic activity in the skeletal muscle, which was attenuated by ET. The changes in LDH activity were more evident in the plantaris muscles of the AS-UN group, most likely because this muscle has a higher frequency of Type II fibers (glycolytic) than the soleus muscle. The increased percentage of Type I fibers (oxidative metabolism) in the soleus and Type IIA fibers (more oxidative compared with the IID and IIB fiber types) in the plantaris muscle in the AS-ET group was further evidence that ET attenuated the decrease in aerobic metabolism in these muscles, because studies have shown that ET promotes a fiber type transition and a myosin heavy chain content shift from a fast-to-slow twitch [43], [44]. Our findings regarding the fiber-type transition during HF corroborate a report by Moreira et al. [45] that moderate aerobic training increased the number of slow-twitch fibers in the soleus and plantaris muscles. Thus, our data strongly suggest that aerobic ET prevented the modulation of the fiber type toward the more glycolytic metabolic pattern.

There is a striking association between skeletal muscle atrophy and chronic HF [46]. Decreased muscle fiber CSA is a major aggravator in HF syndrome and has been observed in experimental HF studies [8], [47], [48]. However, the atrophy is dependent on the model used and the type of muscle examined. Oxidative muscles (e.g., the soleus) are more susceptible to damage caused by inactivity or unloading, whereas glycolytic muscles (e.g., the plantaris) tend to be more affected in diseased conditions [49], [50]. Therefore, we decided to evaluate both the soleus and plantaris muscles because of their contrasting metabolic properties. Soleus atrophy in the AS-UN rats was correlated with a reduced CSA of Type I and Type IIA muscle fibers. These findings are consistent with a previous study by Moreira et al. [45], who also observed a decrease in both fiber types (I and IIA) in the soleus muscle. Moreover, in the plantaris muscle Moreira et al. [45] observed atrophy only in the glycolytic-type fibers, similarly to our results, which the plantaris muscle presented only IID and IIB glycolytic fiber-type atrophy in the AS-UN rats. This result suggests that only the glycolytic fiber types were more damaged under HF conditions and might exhibit a greater loss of blood perfusion, and increased damage from reactive oxygen species and local inflammation, compared with oxidative fiber types [34], [39], [51].

Abnormalities in HF affect various endocrine systems leading to an imbalance of catabolic and anabolic function. Skeletal muscle atrophy during HF may be induced by the activation of local and systemic markers of inflammation, most notably inflammatory cytokine tumor necrosis factor (TNF-α), a proinflammatory secreted cytokine that was originally called “cachectin” [52]. In this study, similar to others [53], [54], a significant increase in TNF-α level were observed in the serum and in the tissue in our AS-UN animals, while in the AS-ET animals, exercise attenuated the expression of this cytokine. An anti-inflammatory effect of physical exercise was recently proposed [55]; this effect may be mediated not only by a reduction in visceral fat mass, with a subsequent decrease in the production and release of proinflammatory cytokines such as IL-6 and TNF-α, but also by the induction of an anti-inflammatory environment with each bout of exercise [55]. Thus, our ET program in AS rats may have increased anti-inflammatory cytokines in skeletal muscle, indicating a potential mechanism for the anti-inflammatory effects of aerobic exercise in cardiovascular disease.

Since TNF-α exhibit catabolic effects in various tissues [56], the increased muscular expression of proinflammatory cytokine might further contribute to the local catabolic state with progressive atrophic alterations of the skeletal muscle in HF. The effects of TNF-α on HF-related muscle myopathy are mediated through the activation of a family of transcription factors known as nuclear factor kappa B (NFκB), which regulate ubiquitin-dependent proteosomal system activity (PDU) [57]. NFκB (p50/p65) activation leads to increased expression of the atrogenes MuRF1 and MAFbx [57], which results in sarcomeric protein proteolysis, promoting muscle atrophy. In our study, although there were slight variations in the gene and/or protein expression levels of NFκB, MuRF1, and MAFbx, the increased levels of these catabolic components in the AS-UN group may be at least partially responsible for the atrophy of the plantaris and soleus muscles. Indeed, we previously demonstrated marked muscle atrophy and a reciprocal increase in the components of the ubiquitin-proteasome system (e.g., MuRF1 and MAFbx) in rats in which HF was induced by monocrotaline [58]. These results indicate the possible involvement of these atrogenes in the HF-induced muscle atrophy process. Although several studies have investigated the muscle atrophy that occurs during HF [40], [45], [58], the effects of ET on the atrogene components have not been completely defined. The present finding that aerobic ET performed during the transition from cardiac dysfunction to HF prevents the overactivation of some proteasomal components is similar to that of Gielen et al. [59] that observed a attenuation of MuRF1 by exercise training and Campos et al. [6], who also observed a reduction of ubiquitin-proteasome system activation in response to ET. Campos et al. [6] also indicated that the possible attenuation of the ubiquitin-proteasome system by ET may be due to improvements in the redox balance driven by the increase in antioxidant enzymes and reduced levels of inflammatory cytokines, corroborating our finding of a decrease in TNF-α levels [4], [6], [54].

The transforming growth factor-β-related protein myostatin, a key regulator of muscle growth, has been considered an important mediator of cardiac-induced skeletal muscle wasting and cachexia in animal studies [5], [60]. However, the effects of ET on myostatin expression in skeletal muscle remain inconclusive. Studies using swimming endurance training have shown a reduction in the myostatin mRNA content in skeletal muscle [61], [62]. Lenk et al. [17] observed an increase in myostatin levels in the myocardium and the gastrocnemius muscle in a rat model of ischemic cardiomyopathy, and ET on a treadmill led to a significant reduction in myostatin protein expression in the skeletal muscle and myocardium of HF animals. Our results support these findings; we observed a significant increase in myostatin expression in the soleus and plantaris muscles in the AS-UN group and aerobic ET prevented the increase in myostatin expression in the AS-ET group in both muscles evaluated. Similar to Lenk et al. [17], we also demonstrated that aerobic ET decreased the expression of TNF-α. The significant reduction in myostatin levels in the soleus and plantaris muscles after ET could be linked to the reduction in the levels of the TNF-α; however, further experiments investigating TNF-α levels and myostatin should be performed to confirm the findings of the present study.

In chronic HF, alterations in multiple anabolic and catabolic systems result in progressive catabolism, which leads to cardiac cachexia in advanced stages of the disease [3]. As a key regulator of normal muscle growth and hypertrophy, the role of the IGF-I/AKT/mTOR signaling pathway has recently received increased attention as a potential means of suppressing protein breakdown while promoting muscle growth [9]. In patients with chronic HF [7], [37] and in animal models of chronic left-ventricular dysfunction [10], [63], reduced skeletal muscle expression of IGF-I is associated with muscle atrophy. In addition, in advanced stages of HF have been described low serum levels of IGF-I due to a growth hormone resistance and a loss of lean muscle mass [3]. In the present study, we observed low serum levels of IGF-I in AS-UN rats compared to Sham-UN, without difference in AS-ET vs. Sham-ET animals. In contrast, the tissue concentration of IGF-I was similar among the groups, indicating that AS-induced HF did not downregulate the IGF-I/AKT/mTOR pathway locally, and suggesting that the although aerobic ET counteracted muscle catabolism, aerobic ET did not enhance the muscle anabolic state. Although disease progression from mild-moderate to severe HF is likely multifactorial and is poorly understood, the available data suggest that GH/IGF-I may be implicated in this pathology. A possible explanation for these findings was suggested by Arcopinto et al. [64], who stated that during the gradual development of HF the IGF-I values are normal or slightly elevated in the beginning; in the intermediate stage, IGF-I levels tend to decrease or remain unaltered; and in advanced stages, IGF-I reduces and a markedly increase in GH circulating levels occur. Furthermore, the changes in skeletal muscle hypertrophy and atrophy turnover do not always proceed according to the balance between protein breakdown and protein synthesis [65]. Analyses of rat muscle growth by Li et al. [66] demonstrated that starvation causes decreased protein synthesis and increased protein degradation in both fast and slow rat muscles. However, in a model of muscle denervation, the authors observed an increase in protein degradation and an increase rather than decrease in protein synthesis [67]. Taken together, it is possible that in our experiment, the stage of HF greatly influenced the catabolic components without any change in the anabolic pathway and that the prevention of skeletal muscle atrophy by ET occurred independent of protein synthesis.

The members of the forkhead box O (FoxO) family are transcription factors responsible for the cross-talk between protein degradation and protein synthesis [9]. Similar to Reed et al. [68], who demonstrated that FoxO transcriptional activity is increased in skeletal muscles under 2 cachectic conditions, we also observed that HF-induced muscle atrophy is associated with an increase in the expression of FoxO in both muscles studied. By contrast, aerobic ET attenuated this change in FoxO expression; specifically, the effects of aerobic ET in phosphorylate FoxO1, contributing, at least in part, to the prevention of skeletal muscle atrophy. Here, we extend our findings to the preventive effect of aerobic ET on FoxO inhibition. FoxO activity is modulated by other intracellular signaling molecules, such as peroxisome proliferator-activated receptor gamma coactivator 1 alpha (PGC1α), a cofactor involved in mitochondrial biogenesis, fiber-type switching, and angiogenesis [69], [70]. Low levels of PGC1α have been reported to contribute to muscle wasting during chronic HF [71]. We observed a decrease in the PGC1α levels only in the plantaris muscle, although HF-induced atrophy was observed in the soleus and plantaris muscles of the AS-UN animals. Considering that glycolytic muscles are more vulnerable to catabolic muscle wasting than are oxidative muscles [51], [72] this result demonstrates the distinct responses to HF stimuli in the soleus and plantaris muscles, which in this case might be explained by 2 main factors. The first is a decrease in blood supply, which was likely more pronounced in the plantaris muscle than in the soleus muscle. This difference could occur because the plantaris muscle contains glycolytic fiber types, which receive less blood and, consequently, reduced antioxidant defense [71]. The second factor could be related to fiber-type modulation. The decrease in the percentage of type IIA and increase in the IID fibers in the plantaris muscle of AS-UN animals in our study, in association with a decrease in the PGC1α content, supports previous findings of Russell et al. [73], who showed that PGC-1 protein levels were higher in the type IIA fibers, lower in the type I fibers, and lowest in the type IID/X fibers in human skeletal muscle, thereby suggesting the idea that the increase in the IID fiber types in plantaris muscle in our experiment possibly induced the reduction in the PGC1α content of AS-UN animals. By contrast, the absence of changes in PGC1α levels in the soleus muscle could be related to the Type I-to-IIA fiber conversion, which occurred within fiber population in which PGC1α levels is already high [71], [73]. In addition, although our ET did not altered PGC1α levels in Sham-ET group, moderate and intense aerobic ET is known to promote an oxidative phenotype, stimulates PGC1α expression in skeletal muscle [74], and by PGC1α overexpression prevents muscle atrophy through the inhibition of the transcriptional activity of the FoxO3, NFκB, and myostatin signaling pathways [9], [75]. In the current study, we observed that aerobic ET during the transition from cardiac dysfunction to HF prevented the reduction in PGC1α expression levels in the plantaris muscle. Of particular interest, the expression levels of PGC1α and FoxO1 in this muscle were negatively correlated (mRNA, *r* = −0.69, p = 0.003; protein, *r* = −0.90, p<0.0001). Although these results do not demonstrate a causal relationship between the anti-proteolytic component PGC1α and the atrophic factor FoxO1 in AS-induced HF, they confirm previous reports demonstrating a strong negative correlation between these proteins and provide insights into the functional role of PGC1α in the skeletal muscle in protection against catabolic muscle wasting [14], [71], [75].

Although much has already been achieved, there is a great deal more that we need to learn about ET applied during HF. Aerobic exercise training is now an integral part of the recommended treatment for stable New York Heart Association (NYHA) class I–III HF patients [76]. Nevertheless, the understanding of the molecular differences between beneficial vs. ‘maladaptive’ eccentric cardiac growth will instruct potential future therapy. Also, the precise definition of the molecular and cellular mechanisms mediating the beneficial effects of ET during HF should be elucidated to devise optimal treatment strategies that could in theory beneficially impact cardiovascular diseases and HF.

## Limitations

Although the ascending AS is one of the more widely used surgical methods to induce pressure overload in rats [22], [47], [77], [78] a limitation of this model is the evaluation of the grade of AS that could be done by the analyze of the aorta flow velocity. As this measurement is not reproducible technically in supravalvar AS in rats, this issue can be partially solved by using the left ventricular systolic pressure data [79] or by echocardiography parameters [18], as we used in the present study.

The beneficial effects of aerobic ET protocol attenuating the progression of the disease were evidenced in cardiac structure and function parameters. However, we cannot conclude whether the aerobic ET affected the skeletal muscles directly, or indirectly preventing the deterioration from cardiac dysfunction to HF. In addition, although we measured the degree of exercise tolerance and performance, we did not make a direct assessment of skeletal muscle force.

Finally, we used NFκB (p65) from nuclear and cytosolic fraction for assessment of ubiquitin proteosomal system activity (PDU). This protein does not allow us to verify the correct activity of the NFκB, but taken together with others downstream protein expression, this assay provides an important indicator of the PDU involvement in skeletal muscle during the transition from cardiac dysfunction to HF.

## Conclusions

Collectively, our data provide evidence that aerobic ET performed during the transition from cardiac dysfunction to HF has beneficial effects on cardiac structure and function. Our results also show that muscle atrophy observed in HF due to an increase in catabolic pathways, was prevented by aerobic ET not via an increase in anabolic factors but by counteracting the catabolic activity, presumably by PGC1α expression.
